# Graphene Oxide–Protein-Based Scaffolds for Tissue Engineering: Recent Advances and Applications

**DOI:** 10.3390/polym14051032

**Published:** 2022-03-04

**Authors:** Elena Iuliana Biru, Madalina Ioana Necolau, Adriana Zainea, Horia Iovu

**Affiliations:** 1 Advanced Polymer Materials Group, Department of Bioresources and Polymer Science, University Politehnica of Bucharest, 1-7 Gh. Polizu Street, 011061 Bucharest, Romania; iuliana.biru@upb.ro (E.I.B.); madalina.necolau@upb.ro (M.I.N.); adriana.zainea@upb.ro (A.Z.); 2 Academy of Romanian Scientists, 54 Splaiul Independentei Street, 050094 Bucharest, Romania

**Keywords:** protein stability, graphene oxide interactions, reinforced scaffold, regenerative medicine

## Abstract

The field of tissue engineering is constantly evolving as it aims to develop bioengineered and functional tissues and organs for repair or replacement. Due to their large surface area and ability to interact with proteins and peptides, graphene oxides offer valuable physiochemical and biological features for biomedical applications and have been successfully employed for optimizing scaffold architectures for a wide range of organs, from the skin to cardiac tissue. This review critically focuses on opportunities to employ protein–graphene oxide structures either as nanocomposites or as biocomplexes and highlights the effects of carbonaceous nanostructures on protein conformation and structural stability for applications in tissue engineering and regenerative medicine. Herein, recent applications and the biological activity of nanocomposite bioconjugates are analyzed with respect to cell viability and proliferation, along with the ability of these constructs to sustain the formation of new and functional tissue. Novel strategies and approaches based on stem cell therapy, as well as the involvement of the extracellular matrix in the design of smart nanoplatforms, are discussed.

## 1. Introduction

Tissue engineering (also called regenerative medicine) comprises the efforts to create functional human tissue from the appropriate cells with the aim to cure, not merely treat, by repairing or replacing tissues or organs that fail due to illness or malfunction, genetic malformations, congenital abnormalities, or wounds and injuries. Furthermore, surgical resection performed for tumor removal frequently requires reconfiguration and reestablishment of the lost structure, and techniques employing transplantation of auto- or allografts are limited by donor site morbidity, poor immunogenicity, and graft restoration [1].

The success of tissue engineering relies on four main factors that are involved in the repair, maintenance, or restoration process of the damaged tissue: (i) the use of appropriate cells that are able to regenerate or replace tissue; (ii) a suitable environment, such as scaffolds to support cells; (iii) the appropriate biomolecules, such as growth factors able to produce healthy and productive cells; and (iv) the mechanical forces in the physical microenvironment to stimulate the development of new tissue cells. The cells can be directly collected from the target organ, developed from precursor or stem cells, or harvested from cell lines developed in the laboratory or, preferably, from the affected patient to limit complications regarding rejection reactions.

For replication or restoration, modern procedures are employed that involve the use of multidisciplinary knowledge of cell biology, immunology, materials chemistry and biomaterials engineering, preclinical studies, etc. In the last years, several approaches have been employed to mitigate the lack of donor tissues and organs.

Supporting scaffolds can be obtained from donor tissue or natural or synthetic polymers that may replicate the strength or endurance of native organs or tissues. There is a significant shortage of donated tissues and organs due to the increasingly aging population [2], and the immense demand for organ and tissue transplants has generated endless research studies on the rejuvenating features of human cells.

Since the native physiological microenvironment has the ability to be locally adapted for the regenerative process, multiple biomaterials have been explored to allow the infiltration, division, and differentiation of implanted cells. Scaffolds are required to mimic the specific tissue cellular microenvironment in order to support cell growth, differentiation, and proliferation and to provide an appropriate physiological morphology and the possibility of co-culturing various cells. From a mechanical point of view, scaffolds provide mechanical and shape stability to the repaired tissue. From a biological perspective, scaffolds are architectures that sustain the development of the extracellular matrix (ECM) and cell establishment. In addition, the permeability of the reconstructed tissue is a key element for enabling nutrient transfer from culture media and supports the elimination of noxious secondary products from the material without adversely affecting culture conditions. Further, the new scaffold should be stable for a certain period of time to enable the damaged tissue to repair or regain the ability to be restored. Time-dependent biodegradability is another important aspect to consider for tissue scaffolds in order to allow the take-over of cells to promote the healing process.

Polymeric scaffolds are desirable because of their high water-holding capacity and structural similarity to the ECM [3]. In the last years, both synthetic and natural polymers have been employed for tissue engineering, and scaffold features have been proved to depend on the polymer structure and concentration, pore size, flexibility, stiffness, etc. Synthetic polymers such as polylactic acid (PLA) [4], polyvinyl alcohol (PVA) [5], poly (lactic-co-glycolic) (PLGA) [6], and poly ε-caprolactone (PCL) [7] have been used for the preparation of 3D scaffolds due to their easily adjustable porosity, mechanical performance, and degradation time. With their higher biocompatibility, natural polymers such as gelatin [8], collagen [9], chitosan [10], alginate [11], elastin [12], and fibrin [13] have attracted researchers’ attention for the preparation of 3D scaffolds that faithfully replicate the native tissue vasculature and channel interconnections that allow the perfusion of nutrients and oxygen diffusion during regeneration. Moreover, the degradation kinetics can be coordinated with the regenerative effects in order to drive the reformation of scarless tissue, reducing the necessity for secondary surgical procedures to eradicate any degenerated scaffold [14].

Proteins are involved in biological processes and play an essential role in regenerative therapies. They are one of the best candidates for tissue engineering thanks to their superior biocompatibility, biodegradability, and bioresorbable properties [15]. Additionally, their relatively low cost and commercial availability make them attractive for designing advanced functional biomaterials for advanced medical applications, such as gene [16] and drug delivery [17], cellular regenerative medicine [18,19], biosensors [20], and photothermal therapies [21,22].

However, proteins are unstable in non-physiological environments [23], and protein-based scaffolds exhibit limited mechanical properties and undergo rapid degradation in the physiological milieu [1]. To properly engineer functional tissues, new scaffolds need to possess a few key features, such as mediating cell growth and modulation, delivering bioactive compounds, providing appropriate physical and chemical signal cues, and stimulating the mechanical properties of the native tissue [24]. Over the past decades, significant efforts have been dedicated to replicating the mechanical integrity, morphology, and architecture of real natural human tissue, particularly by developing new preparation techniques and reinforcing protein-based scaffolds with nanomaterials that mimic native tissue environments to improve tissue growth, differentiation, proliferation, cellular signaling, etc. The introduction of nanofibers, nanoparticles, nanotubes, or other inorganic carriers [25,26,27,28] was found to be highly efficient for mediating tissue formation.

Carbon nanomaterials have proven to be an excellent platform for the development of 3D tissue engineered scaffolds due to their compatibility with the natural ECM and their extraordinary mechanical strength and conductive properties [29]. Various carbon allotropes, such as nanodiamond, fullerenes, carbon nanotubes, and carbon nanofibers, have been successfully employed to mitigate the mechanical limitations of natural polymer-based scaffolds and to create the required porosity without altering the biological properties of the tissue [30,31,32]. From the carbon family, graphene and its derivatives have been the most extensively studied nanomaterials due to their exceptional versatility in terms of physical, chemical, and biological behavior. Currently, graphene and its derivatives are widely investigated for biomedical applications such as drug delivery [33], tumor therapy [34], and theranostics [35,36]. In the area of biomedical applications, biocompatibility and biosafety represent key factors of the chosen materials. While the toxicity of graphene nanomaterials is still disputed [37], studies have shown that their biosafety is related to the layer shape, dimensions, and surface functional decorations [38,39,40]. Although graphene nanostructures have received enormous attention in tissue engineering applications, most of the reviews have focused on the introduction of preparation techniques and general biomedical applications. The influence of graphene derivatives on protein structure and biological activity has rarely been summarized. This review explains the properties of various proteins that have been used for tissue engineering and describes the possible interactions of graphene nanostructures in terms of protein structure, stability, and biological activity and the utilization of graphene oxide (GO)–protein complexes for tissue engineering. Furthermore, this review provides fundamental insight into protein dynamics in the presence of graphene derivatives that will contribute to designing advanced functional biomaterials for regenerative medicine.

## 2. Proteins for Tissue Engineering

The human body is still unable to regenerate tissues in extensive defects because of the lack of ECM to fill them in cells, tissues, and organs. The use of biomaterials, such as proteins, promotes this extracellular environment; they have a significant impact on tissue regeneration due to their intrinsic bioactivities, which include cell proliferation, adhesion, and biocompatibility with native healthy tissue [41]. In this sense, a traditional approach, such as the fabrication of polymer scaffolds that can act as supports for cell anchorage and can be paired with proteins that have certain properties, particularly biocompatibility, may lead to the creation of biocompatible biomaterials that can mimic regular ECM [42]. In this section, the main protein types involved in regenerative medicine applications are described.

### 2.1. Collagen and Gelatin

Collagen is the primary structural component of the organic component of the bone ECM. The structural complexity of the collagen molecule results in the unique properties of type I collagen. It is a crosslinked, extremely dense collagen that provides mechanical and chemical stability to the bones. Collagen fibers are a heterogeneous mix of different structures; however, type I collagen predominates in bones. It is made up of three polypeptide chains, each of which is a left-handed helix. Direct inter-chain hydrogen bonds connect these chains, limiting rotation and ensuring the stability of the triple-helix collagen [43]. Because collagen is the most abundant structural protein in vertebrates and plays important roles in controlling cell functions such as cell adhesion, proliferation, and differentiation, collagen-based biomaterials have been extensively used to prepare porous scaffolds for engineering tissue [44]. Furthermore, collagen type I–based biomaterials and fiber processing techniques have been employed to mimic the natural microstructure of various tissues; thus, porous collagen scaffolds are being used in the tissue engineering of cartilage, meniscus, bone, ligament, nerve, skin, and related structures [45,46]. Despite its high biocompatibility, collagen exhibits poor physical and chemical stability, which includes low mechanical strength, increased sensitivity to enzymatic degradation, and low thermal stability.

Gelatin is a fibrous protein extracted from denatured native collagen, and its structure, composition, and biological potential resemble those of native collagen. Individually, or together with natural or synthetic polymers, gelatin has been extensively used for the assembly of stable in vitro bioactive scaffolds for tissue engineering. It also improves bone cell adhesion and migration due to Arg-Gly-Asp sequences [47]. Samadian et al. demonstrated in vivo that gelatin can stimulate neo-bone formation, osteocytes in lacuna woven bone formation, and angiogenesis in the defect position [48]. The fact that gelatin is a polypeptide with a high molecular weight and derived from the thermal denaturation of collagen makes it not only less expensive than collagen but also easier to obtain from concentrated solutions and less immunogenic. Nevertheless, the main disadvantage is that it has unsatisfactory mechanical properties. For example, in comparison to the mechanical characteristics of bone, it has a low tensile strength, a high compressive strength, and a rather low shear stress strength. Gelatin has shown a low elastic modulus as well [49].

### 2.2. Fibronectin

Fibronectin is involved in several stages of wound healing, the most important being cellular adhesion. Other functions include cell growth and migration mediation. Fibronectin is a significant glycoprotein encountered in all tissues and is required for many diverse cell–matrix interactions. Depending on the origin, this protein is subdivided into: plasma fibronectin, which is produced in a soluble form by hepatocytes in the blood plasma, and cellular or tissue fibronectin, which is produced by multiple cells, such as fibroblasts, endothelial cells, and keratinocytes [50]. As a constituent of the ECM, fibronectin is also identified in the initial stages of bone formation. Its presence in areas of skeleton genesis interferes with cell adhesion and facilitates osteoblast differentiation to develop mineralized nodules [51].

### 2.3. Elastin

Elastin is the main mammalian extracellular matrix protein that gives tissues elasticity and is found in the arteries, lungs, skin, elastic ligaments, bladder, and elastic cartilage. One of its functions is mediating cell interactions, such as dermal fibroblast attachment and spreading via integrin V3. Moreover, as a chemoattractant, it attracts smooth muscle cells, endothelial cells, and monocytes. Elastin is essentially derived from elastin-rich bovine and porcine tissues. Purified elastin, on the other hand, is insoluble because it is crosslinked, making it harder to manipulate as a biomaterial. Hydrolyzed elastin procedures improve solubility but contain heterogeneously fragmented elastin, which can suffer structural integrity and cellular signaling ability losses [52]. Elastin is a highly elastic protein that is essential for cartilage tissue’s proper function due to its contribution to suppressing compressive loads and assimilating mechanical forces acting on articular joints [53]. The three hydrophobic sites of lysine residues in the elastin molecule are mainly associated with the crosslinking process. These three regions adjacent to the two crosslinking domains are able to sustain self-assembly. The main disadvantage of elastin, though, is that its purification is quite challenging for tissue engineering applications. In addition, the low mechanical properties result in a lack of migration and adherence to the cell [54].

### 2.4. Human Serum Albumin

This heart-shaped globular protein has six helical subdomains that repeat and is composed of 67% helices, 23% random coils, 10% twists, and no sheets. HSA includes three major domains, according to high-resolution X-ray crystallographic structures, and is the most abundant protein in plasma, accounting for approximately 60% of total plasma proteins, and it is known for being responsible for several functions. Some of these include maintaining colloid osmotic pressure and binding and transporting a wide range of ligands, including fatty acids, bilirubin, hormones, metal ions, and medicines [55,56]. An important application for this protein is also found in cell differentiation, especially in recent years, and significant studies have been carried out on processes such as the regulation of osteogenic differentiation [57].

### 2.5. Bovine Serum Albumin (BSA)

BSA, a protein that resembles HSA, is formed by a single polypeptide chain of 583 amino acid residues. The structure is secured by the crosslinking of 17 disulfide bridges of cysteine (Cys) amino acid residues [58]. This physiological protein is frequently employed as an effective vehicle in tissue engineering and drug delivery systems due to its biodegradability, non-antigenicity, non-toxicity, and easy production [59].

Because of its high water solubility and low cost, it is widely employed in the pharmaceutical sector and tissue engineering. Furthermore, it has been widely employed as a protective agent for several growth factors [60].

### 2.6. Laminins

Laminins self-polymerize into a nascent scaffold via their N-terminal domains; the scaffold is then cross-coupled with other constituent parts, such as collagen IV, integrins, nidogens, agrin, and perlecan, to form the basement membrane. The basement membrane serves as an adhesion substrate for tissue homeostasis [61]. An important aspect of laminins is that they appear to mediate cellular functions such as adhesion, migration, growth, differentiation, and apoptosis via interactions with particular cell surface receptors, such as integrins, dystroglycan, or sulfated glycolipids [62]. To date, 16 different laminin isoforms have been found, with distinctive expression patterns across tissue types and developmental stages [63]. Since this protein has various isoforms in each tissue, incorporating an integrated laminin isoform into the scaffold has been considered a strategy for overcoming the artificial matrix’s diversity [64]. In addition, small peptide sequences derived from laminin have been extensively investigated as an alternative to full-length laminin for conferring bioactivity to 3D matrices. Moreover, among their several advantages, laminins exhibit the ability to be chemically synthesized on a wide scale, greater resistance to denaturation and enzymatic degradation, the capacity to be integrated at increased densities to achieve amounts comparable to the native protein and a reduced risk of causing immune rejection [63].

### 2.7. Growth Factors

Bone morphogenetic proteins (BMPs) are a group of growth factors and cytokines that were first characterized by their capability to trigger bone and cartilage formation, but they are now regarded as a group of major morphogenetic signals that organize the tissue architecture of the entire body [65]. All BMPs are basic proteins with an isoelectric point (pI) between 7.7 and 9, while BMP-2 and BMP-7 have identical pIs at 8.2 and 8.1, respectively. In addition, they have a lot of hydrophobic sites on their surface. Therefore, they show limited solubility at physiological pH, which is believed to be related to their pharmacological activity. Rapid clearance is another characteristic of BMPs. For example, BMP-2, when administered in buffer alone, has a half-life of ~7 min in non-human primates [66]. Despite these relevant data, large-scale studies have confirmed the relatively high incidence of adverse events related to the clinical use of BMP-2, including life-threatening cervical edema. In fact, the Food and Drug Administration (FDA) has issued a warning about potential life-threatening complications of BMP-2. Adverse events associated with BMP-2 are not only frequent but also sometimes catastrophic, especially with anterior cervical fusion. These include swelling of the cervical and soft tissues, airway obstruction, and the need for re-operation [67].

The fibroblast growth factor (FGF) family of proteins comprises signaling proteins secreted by tissues to regulate cell metabolism, proliferation, differentiation, and survival. The above-mentioned proteins bind to heparin and have a wide range of mitogenic and angiogenic activities, including the regulation of normal cell proliferation in epithelium, bone, soft connective tissue, and nervous tissue [68]. In particular, FGF-2 has been shown to play a key role in angiogenesis, wound healing, embryogenesis, and nerve survival and differentiation, and it has multifaceted effects on various tissues and organs. Therefore, signaling of the FGF-2 receptor (FGFR) by bioactive biomaterials can have many important uses, from the treatment of myocardial infarction to muscle regeneration and spinal cord injury [69].

Decellularized extracellular matrix (dECM) is derived from two main sources: one from native tissues and body organs and the other from organs made from regenerated tissues and cultured cells. Thus, dECM is generally classified as tissue-derived dECM and cultured cell-derived dECM. Each method has several advantages and disadvantages. For tissue/organ-derived dECM, the similarity to the composition and structure of native ECM represents an asset. The limitations, though, include the source of ECM, the difficulty of using it in large-scale in vitro assays, and large-scale variations due to cancer variety. With regard to cultured cell-derived dECM, the advantage is the possibility for large-scale in vitro assays. The downfall, however, is the difficulty in preparing dECM that fully simulates the composition and structure of the local ECM [70]. One of its main applications is found in tissue engineering, namely, dECM scaffolds, referring to biological material formed from human or animal organs/tissues, which have the shortcoming of needing to remove immunogenic cellular components by employing cellularized technologies. According to ECM sources, dECM scaffolds are categorized into three categories, namely, autogenous dECM, allogenic dECM, and genogenic dECM. Because autogenous ECM scaffolds encounter tissue limitations and surgical complications, most ECM scaffolds come from allogeneic or xenogenic donor tissue. However, donor site morbidity, differences in architecture and mass composition, and immunogenicity problems produced by incomplete cellularization may occur in the allogeneic/xenogenic dECM [71].

In addition to proteins, peptides are significant modifiers of the polymer backbone. Thus, peptides have more advantages than proteins, which is an essential aspect. Peptide production, for example, is less complicated and less expensive than the synthesis of a full-length protein. Similarly, modifying peptides is considerably simpler than modifying high-molecular-weight proteins. Furthermore, because of their low immunogenicity, peptides are more tolerant to environmental conditions (e.g., pH and temperature). Because of these aspects, several peptides that mimic the functions of ECM proteins and some growth factors have been employed [42].

The challenging issue with the use of peptides is the fact that there is a need to improve the biological activity of implantable materials because they can only address one biological target. Combining peptide sequences with synergistic and perhaps complementary effects, allowing for the treatment of two or more biological effects, is one way to improve the performance of peptide molecules. Multifunctionality can hence be imparted to biomaterials without the use of proteins. Thus, this strategy may be a way to capitalize on the benefits of native ECM proteins while avoiding their well-documented drawbacks. Under these circumstances, the functionalization of biomaterials by combining peptides has grown in significance in the field of tissue engineering in recent years [72].

In the last years, several studies have established that GO–protein conjugates exhibit a handful of advantages over free proteins, including unique biological and chemical properties, increased biocompatibility and stability in biological environments, the ability to conjugate to target receptors or sites, and cell internalization [73]. The appropriate function of proteins for regenerative medicine depends on their secondary and tertiary structural integrity. Physical and chemical interactions with carbon nanostructures in ambient conditions may disrupt the structural integrity, leading to denaturation or misfolding of the protein chain, which may initiate protein-mediated diseases and abnormalities [15,74,75].

## 3. Graphene Oxide–Protein Interactions

Graphene represents a crystalline monolayer of carbon atoms that are densely assembled in a honeycomb-like architecture. Its extraordinary two-dimensional (2D) structure is entirely made of sp2 hybridized carbon atoms and out-of-plane π bonds that can bind with adjacent atoms [76]. With a uniform electron distribution, this unique atomic structure is an exciting material with high thermal and electrical conductivity [77], exceptional mechanical properties [78], optical transparency [79], chemical stability, and an extremely large surface area [80]. Since its first isolation in 2004 [81], graphene has been intensively studied for biomedical applications, which have shown that it can interact with biomolecules such as DNA, proteins, enzymes, or peptides for the preparation of 3D scaffolds involved in tissue regeneration. Although several approaches, such as chemical vapor deposition [82], mechanical exfoliation [83], and electrochemical exfoliation [84], have been established, the synthesis of single-layer graphene (SLG) remains difficult and costly. Moreover, graphene is highly hydrophilic and tends to agglomerate, hindering its dispersion in aqueous solutions or non-toxic solvents.

Graphene oxide (GO), on the other hand, is the oxidized form of chemically modified SLG, and it is obtained through the oxidation treatment of graphite and ultrasonic exfoliation [85], after which its surface contains various oxidized species, such as hydroxyl (–OH), carboxyl (–COOH), and epoxide groups (C–O–C), thus facilitating the dispersion of the basal layers of graphene in water. The carboxyl groups situated at the edges of the graphene layer induce colloidal stability and pH-dependent negative surface charge. The hydroxyl and epoxide functional groups, located at the surface of the graphene plane, are uncharged but polar, forming weak interactions, hydrogen bonding, and other side reactions [86]. Additionally, these reactive oxygen sites of GO allow further chemical functionalization with biomolecules such as proteins and polysaccharides, which is a major advantage for bio-related applications. The basal plane of GO still comprises free surface π electrons from the unaltered areas of graphene that are hydrophobic and able to participate in π–π interactions for drug loading and non-covalent functionalization [87]. Therefore, GO is an amphiphilic structure, and the presence of functional groups creates structural defects that reduce the mechanical, electrical, and thermal properties compared to unmodified graphene.

In an attempt to restore the sp^2^ hybridization structure, GO has been submitted to reducing treatments that form reduced graphene oxide (rGO). rGO can be produced by applying thermal, chemical, or UV reduction processes to GO using harsh chemical agents. rGO is generally produced to re-establish the electrical and thermal conductivity of the material by removing the oxygen functional groups, surface charge, and hydrophilicity [88]. However, most of the reducing agents (such as hydrazine [89]) are toxic, making rGO unsuitable for biomedical applications. In recent studies, less toxic protocols have been developed involving ascorbic acid [90,91] as a reducing reagent, showing higher biocompatibility for the obtained rGO compared to hydrazine reducing methods. Although hydrophobic, the removal of oxygen species from the GO structure is only partially achieved, and rGO still possesses a low content of oxidized functional groups that will enable the aqueous suspension of graphene-like layers. In addition, several studies have shown that rGO is biocompatible and stable for cell culture [92], drug delivery [93], tissue engineering [94], and other biomedical applications.

Protein–GO/rGO-based scaffolds possess improved mechanical properties resulting from the combination of the extremely high specific surface area of graphene sheets and their strong interactions with the protein chains. It was previously demonstrated that the dimensions, shape, and decorated surface of graphene derivatives play a significant role in determining the interactions with biologically active molecules. Both covalent and non-covalent binding, such as electrostatic, van der Waals, hydrogen bonding, hydrophobic/hydrophilic interactions, may occur and strongly affect the secondary and tertiary structures of proteins, which may cause the loss of structural integrity and lead to misfolding [95].

**Figure 1:**
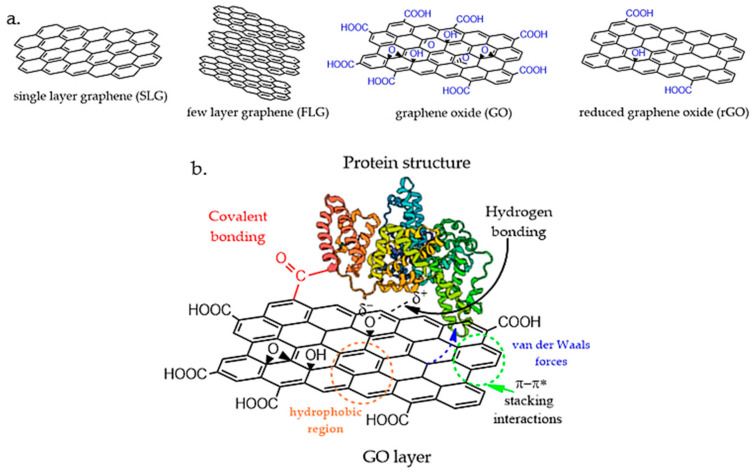
(**a**) Structures of graphene-derived nanomaterials; (**b**) illustration of possible covalent/non-covalent interactions between GO and protein structures.

The interfacial interactions between GO/rGO layers and protein structures, as illustrated in Figure 1, are influenced by two main parameters: (i) the distribution and physical alignment of the protein on graphene layers; (ii) the affinity of the GO/rGO structures to bind with the protein backbone and functional groups. The entire architecture of protein–GO/rGO is dependent on the chemical interactions between the protein and GO/rGO nanostructures that affect the final mechanical stability, morphology, and porosity, as well as degradation kinetics. Graphene-based nanomaterials exhibit a high tendency to aggregate due to their π–π stacking interactions, and graphene colloids may create non-homogeneous accumulation points and weak network islands that ultimately lead to non-uniform stress distribution within the scaffold.

### 3.1. Non-Covalent Interactions

The physical adsorption of proteins on the surface of GO is achieved through non-covalent interactions. This is one of the most simple and direct methods used for protein scaffold reinforcement. The non-covalent interactions involved in protein–GO/rGO complex formation are electrostatic interactions, hydrogen bonding, hydrophobic interactions, van der Waals forces, and π–π stacking interactions. The non-specific interactions are mainly controlled by the layer content and interfacial stress transfer between the GO/rGO layers and protein structure. Zang et al. [96] studied the binding mechanism of GO with BSA at the molecular level to understand the binding affinity of serum albumins towards the GO structure. In this case, it was observed that hydrophobic forces, hydrogen bonds, van der Waals, and π–π* stacking interactions contributed to the adsorption of BSA onto the GO surface. Through circular dichroism (CD) investigations, it was revealed that the secondary and tertiary structures of the protein are considerably changed in the presence of GO, with a considerable decrease in α-helix content. Additionally, the presence of GO decreased the binding affinity of BSA to drugs and affected the stability of BSA against thermal degradation. In another study [97], Wu and coworkers studied the BSA conformation and adsorption behavior onto the GO surface under various pH conditions, showing that the adsorption mechanism is mainly controlled by the protein conformational change and electrostatic and hydrophobic interactions, and clear secondary structural perturbations were observed upon interaction with the GO surface. In 2020, Hampitak et al. [98] studied BSA interactions and conformations on GO and rGO surfaces using a quartz crystal microbalance with dissipation monitoring (QCM-D) and found significant differences in molecular orientation and conformation, mass adsorption, and biochemical functionality. The predominant forces during the adsorption of BSA onto GO and rGO were shown to be hydrophilic and hydrophobic interactions, respectively. In this case, it was observed that BSA on the GO surface can retain its binding sites while, on the contrary, a denatured ad-layer of BSA forms on rGO, followed by further binding of active BSA molecules, depending on the concentration of the protein.

Surface functionalization of GO layers affects the stability of biologically active molecules. Bai et al. [99] analyzed the influence of GO and rGO structures on the activity and conformation of lysozyme, showing dramatically different effects. While both GO and rGO adsorbed high quantities of lysozyme after incubation, it was noticed that GO seriously inhibited the lysozyme activity and led to the loss of the secondary structure. On the contrary, rGO had nearly no influence on the enzyme activity and, to some extent, increased the α-helix content. Although neither GO nor RGO induced the fibrillation of lysozyme, the study concluded that rGO nanostructures induced higher biocompatibility in lysozyme conjugates than GO. Bera and colleagues [100] further studied the molecular features of the interaction involving hen egg-white lysozyme immobilized on GO, and while no significant changes in protein secondary and tertiary structures were observed, the protein showed reduced thermal stability. Additionally, from molecular dynamic simulation, it was observed that GO binds at the active site of the protein, resulting in reduced activity.

Non-covalent functionalization is predominantly used in peptide or protein scaffolds for tissue engineering due to its advantageous fabrication methods [101] through electrostatic interactions, hydrogen bonding, hydrophobic interactions, van der Waals forces, and π–π stacking interactions. However, based on the above discussion, it was observed that non-covalent adsorption results in weak conjugate stability, reducing the protein activity, whereas covalent conjugation can enhance the stability of the conjugate to heat, pH, storage conditions, and organic solvents [102].

### 3.2. Covalent Attachment

While non-covalent protein–GO/rGO complexes offer the advantages of relatively easy synthesis, mild conditions, and retention of the intrinsic surface properties of the graphene-based scaffold, the covalent binding of the protein structure to GO/rGO layers allows the formation of more compatible complexes and better dispersion of the graphene layers within the protein matrix. Covalent functionalization involves strong binding through the amino acid groups of the protein and modified functional groups of the GO/rGO surface. Su et al. [103] observed that by immobilizing the protease on the GO surface employing glutaraldehyde as a crosslinking agent, the thermostability and reusability of the scaffold were considerably improved compared with the free enzyme and showed good operational stability. Functionalized GO with amine dendrimer structures has been employed for the immobilization of BSA protein [104], showing a slight increase in the stability of BSA in the presence of carboxylated GO compared to aminated GO in terms of α-helix content. Hermanová and colleagues [105] also demonstrated that covalently immobilized lipase on the GO surface exhibits substantially better resistance to heat inactivation in comparison with the free lipase structure and improved thermal stability and solvent tolerance.

Although studies on covalent protein–GO complexes exist, their ability to be employed in existing biological processes and to enable regenerative healing still requires deeper understanding. Nevertheless, the promising results of covalent protein–GO scaffolds and their proven benefits over non-covalent conjugates highlight the important potential of these materials for tissue engineering applications.

## 4. Applications in Tissue Engineering and Regenerative Medicine

Tissue engineering represents an interdisciplinary domain that involves the development and fabrication of new biomimetic materials with a similar structure and configuration to those of the extracellular matrix, the aim of which is to stimulate and sustain the regenerative mechanism through cell proliferation and differentiation in order to create new tissue. The development of new materials with a similar structure and configuration to those of natural tissue has become of great importance in the field of tissue engineering, and thus, GO–protein bioconjugates have recently drawn researchers’ attention as a potential formulation that can successfully fulfill the requirement of regenerative medicine. Table 1 summarizes the latest applications of bionanocomposites based on biomacromolecules and GO in the field of tissue engineering.

### 4.1. Bone Tissue Engineering

Collagen, as the prevalent fibrillar protein in connective tissues, represents the most suitable biopolymer to synthesize scaffolds for the support of osteointegration. Biosynthetic bone grafts require specific mechanical properties that can be obtained with the aid of composite biomaterials.

The synergic effect between collagen and GO has been shown to have good potential for the development of bioengineered bone tissue. GO represents an effective reinforcing agent due to the strengthening effect on the soft collagen scaffold [116] and also due to the biological activity exerted through multiple functional groups, which increases the similarity of the morphology of the nanocomposite to that of natural bone and guides ions through the network to achieve mineralization. A recent study demonstrated that GO represents an important component in a scaffold designed for tissue engineering due to the ability to control cellular behavior [117].

**Figure 2:**
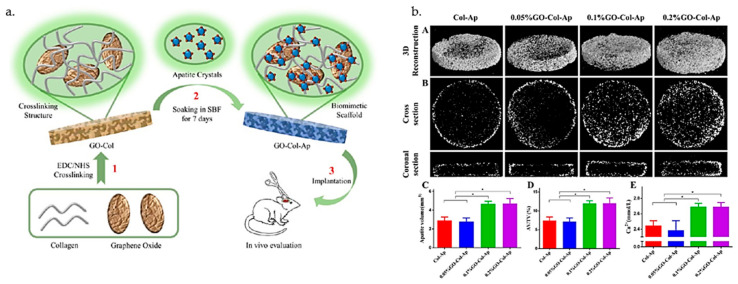
(**a**) Fabrication scheme of GO-Col-Ap scaffolds obtained through chemical crosslinking; (**b**) quantitative analysis and micro-CT images of the scaffolds containing various amounts of GO (* *p* < 0.05). Reprinted with permission from [118]. Copyright 2018, American Chemical Society.

Chuchao Zhou et al. [118] synthesized collagen–GO scaffolds containing apatite (GO-Col-Ap) with increased osteoconductivity and biocompatibility for bone regeneration in cranial defects in rats. The functionalization of the protein with the carbonaceous nanostructure provided multiple active sites for the biomimetic mineralization process due to the numerous oxygen groups present on the basal plane. Composite scaffolds with high porosity and interconnected pores were synthesized through a crosslinking process by using carbodiimide chemistry (EDC/NHS). The deposition of apatite in the scaffold was investigated through micro-CT images, which showed that the apatite mainly attached to the surfaces of the GO–collagen-based scaffolds, rather than within the porous structures (Figure 2).

The authors successfully healed bone defects by implanting biomimetic GO-Col-Ap scaffolds and confirmed the osteoinduction activity of the scaffolds in vivo. Moreover, it was shown that the introduction of GO to porous collagen matrices improved the efficiency of the biomimetic mineralization process. The homogeneous distribution of GO within the collagen scaffold increased the roughness and surface area, which ensured a more favorable environment for cell adhesion, and the hydrophilic functional groups favored the adsorption of nearby proteins, which created favorable microenvironmental conditions for cellular proliferation. Moreover, through the particularities of GO, the biomineralization process was supported by electrostatic interactions, which sustained crystal growth and apatite deposition [119].

Recently, a vascularized tissue engineering chamber was constructed by Fang et al. [120] by employing collagen and GO to promote bone tissue regeneration. This engineered device serves as an in vivo bioreactor [121] that uses mechanical support to sustain a synthetic graft placed inside and also provides adequate microenvironmental conditions for the survival of transplanted cells, thus allowing the regeneration process to evolve. The advantages provided by the combination of collagen with GO in the design of such a device consist of increased biocompatibility along with an advanced ability to protect the bioactive core from macrophage activity. The presence of GO strongly decreased the inflammatory response and promoted osteogenesis, while collagen, as a native protein of bone tissue, supported cellular differentiation and mineralization.

Gelatin, a protein derived from collagen, has been extensively used for the synthesis of bioengineered scaffolds for tissue regeneration with drug delivery capacity [122]. Smart biocomposite scaffolds based on gelatin, hydroxyapatite, and GO loaded with vitamin D were developed by Reza Mahdavi et al. [123]. Morphological and physiological characterization proved that the protein-based scaffold containing 1% GO loaded with vitamin D showed similar characteristics to those of natural spongy bone in terms of density, mechanical properties, and internal structure. The presence of vitamin D promoted the formation of apatite crystals, as well as cell adhesion and proliferation, thus fulfilling the requirements for tissue engineering.

A recent study proposed a fibrous scaffold based on GO and poly (lactic-co-glycolic acid) coated with poly-L-lysine (PLL) as a promising candidate for the restoration of bone defects [124]. The hybrid construct demonstrated not only increased mechanical properties and biocompatibility owing to the carbonic nanomaterial but also high cellular adhesion and proliferation due to peptide chains through the particular electrostatic interaction between the cationic species of the polymeric chains and the negative charge of the cellular membrane. A similar application was targeted in a different study, in which the authors proposed a biomimetic scaffold based on mesenchymal stem cell-secreted extracellular matrix, collagen, and GO for bone tissue engineering of a cranial defect [125]. The nanocomposite hydrogel demonstrated increased osteogenic activity due to the natural occurrence of growth factors in the ECM employed in the synthesis, which also provided multiple sites for cell adherence and proliferation.

Another protein that meets the demands for the synthesis of tissue engineering scaffolds is silk fibroin (SF) on the basis of its high environmental and mechanical stability, low immunogenicity, and increased biocompatibility [126]. The rich content of amino moieties of this protein provides countless possibilities to adjust the chemical configuration through functionalization with different compounds. In addition, the complex chemical structure of this biopolymer allows multiple interactions with GO, resulting in advanced bioconjugates with specific properties for tissue engineering applications [127,128].

Silk is a natural fiber produced by different organisms [129]; thus, it is preferentially used for the development of 3D templates for tissue engineering in the form of mats and nanofibrous scaffolds obtained through the electrospinning technique. Recently, Wu et al. [130] synthesized electrospun SF scaffolds coated with chitosan and bonded GO grafted with bone morphogenetic protein-2 (BMP-2) polypeptide through electrostatic interaction and evaluated the bone regeneration capacity in a skull defect in a rat model. As a result, the nanocomposite scaffold showed increased biocompatibility and cell adhesion and proliferation and specific osteogenic differentiation as a result of the synergistic effect between GO and a BMP-2 polypeptide. Zhang et al. [131] concluded in their study that the concentration and type of GO play a crucial role in determining the final properties of the SF scaffold in terms of fiber diameter, crystallinity, and mechanical properties. Wang et al. [132] produced an injectable stem cell composite hydrogel based on SF and GO and assessed the potential activity of the crosslinked scaffold for bone regeneration. The presence of GO improved the pore structure and mechanical properties of the scaffold and also sustained the growth, proliferation, and osteogenic differentiation of the stem cells.

It has already been demonstrated that nanomaterials such as GO and rGO enhance cellular behavior over various scaffolds in order to promote and sustain tissular regeneration [133]. Fibrin, a fibrous protein, was employed as a biopolymer for the development of a nanocomposite scaffold for bone tissue engineering [134]. Within this formulation, besides GO and fibrin, hydroxyapatite and iron oxide were used in order to obtain a final construct with a similar morphology to that of natural bone. The use of a hydrogel material for the repair of bone defects in this case relies on the ability of the protein to act as a sealing agent that can fill any flaw in the bone matrix, thus encouraging osteogenic differentiation. The final construct showed increased biocompatibility with multiple nucleation sites provided by the presence of the mineral compound and high swelling capacity endowed by the oxygen functional groups of GO, which favor cell adhesion and proliferation.

### 4.2. Cardiac Tissue

When designing scaffolds for cardiac tissue, several parameters, such as mechanical support, conductivity, and highly ordered anisotropic morphology, need to be taken into consideration in order to fabricate functional structures that can imitate the natural extracellular matrix of the heart [135]. Cardiomyocytes are the predominant cells of the heart. These cells are organized in a network and represent the active mechanism that generates contractions and electrical signal propagation in cardiac tissue [136]. Thus, a biomaterial able to maintain a suitable environment for the development of this type of cell will be suitable for the regeneration of the native tissue.

**Figure 3:**
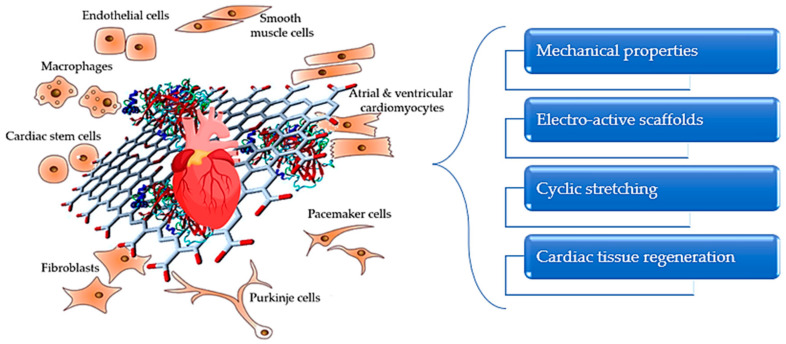
Schematic representation of key features for cardiac tissue regeneration.

GO possesses ideal electrical, mechanical, and biological properties for integration as an active nanomaterial for the development of cardiac scaffolds, as presented in Figure 3 [137,138]. The myocardial extracellular matrix is mainly composed of collagen fibers [139]; thus, biomaterials synthesized from collagen and its derivatives, such as gelatin, can be successfully used for the development of various substrates to enhance cardiac tissue regeneration.

Conductive hydrogel scaffolds based on GelMA embedded with dopamine-doped rGO [140] were recently evaluated as platforms for the fabrication of a functional myocardium layer. The nanocomposite bioconjugate platform exhibited no cytotoxicity in cardiomyocytes and favored the alignment of cells, which started to contract and beat in the same direction. In this formulation, GO plays a crucial role in determining the beat velocity of the newly formed microtissue, along with signal transduction.

Covalent conjugation of collagen with rGO was used for the development of an electroactive cardiac patch. The hybrid formulation favored cardiomyocyte adhesion and exhibited angiogenic properties [141]. A further study using the same biocomplex [142] demonstrated that collagen–rGO scaffolds possess increased mechanical properties and provide a suitable microenvironment for cardiomyocyte coupling, thus increasing cell viability and proliferation.

Zhao et al. [143] fabricated a nanofibrous silk fibroin construct functionalized with a uniform layer of rGO for cardiac tissue engineering with high conductivity and resistance to the mechanical stress caused by the natural dynamic state of the contractile myocardium. In addition, the authors observed that cardiomyocytes seeded on the silk/rGO scaffold exhibited a higher affinity for this substrate in comparison with the silk mesh and spread on the nanofibrous structure along the fibers. This formulation promoted the maturation of the cardiomyocytes, which also developed wide sarcomere structures and led to the formation of functional cardiac tissue with spontaneous beating capacity.

### 4.3. Nerve Tissue

The nervous system is composed of electrically sensitive tissue and represents the control system responsible for the normal function of the entire organism. Thus, the requirements for suitable substitutes in this case are mostly based on the need for a conductive material that can stimulate cell proliferation and organization in functionally connected electrical networks.

GO has been extensively studied in various nanocomposite formulations for such applications on account of its intrinsic electrical and mechanical properties [144].

Silk fibroin, as a versatile protein, was used for the development of composite fibrous scaffolds comprising different loadings of GO and rGO [145] as substrates for neural tissue engineering. The presence of GO in the electroactive formulations did not produce significant morphological modifications in terms of porosity, protein configuration, or fiber diameters. However, the roughness induced by the carbonaceous flakes favored the protein absorption capacity and, at the same time, improved cell adhesion and proliferation in comparison with neat protein-based scaffolds. Ajiteru et al. [146] were the first to develop a 3D printable bioink formulation based on an SF-conjugated rGO structure that, after photocuring, exhibited similar mechanical and electrical properties to those of the spinal tissue. The ability of the bionanocomposite to support the viability and proliferation of Neuro2a cells makes it a suitable candidate for the development of neural tissue engineering platforms.

There have also been several attempts to enhance the electrical properties of silk fibroin/GO bioconjugate. Meng et al. [147] introduced polyaniline, a well-known conductive polymer, to this formulation. The synthesis process of the nanocomposite formulation was based on electrostatic interactions between amino functional groups present in the protein backbone and oxygen groups from the basal plane of GO, followed by in situ polymerization of aniline. The conductivity of the final scaffold was suitable for nerve cell regeneration.

## 5. Advances and Challenges in Tissue Engineering Platforms

Conjugates of GO with biomolecules (proteins or peptides) have emerged as powerful multifunctional platforms for the development of targeted applications in the field of tissue engineering and regenerative medicine owing to their distinctive physiochemical properties and versatile morphology.

**Figure 4:**
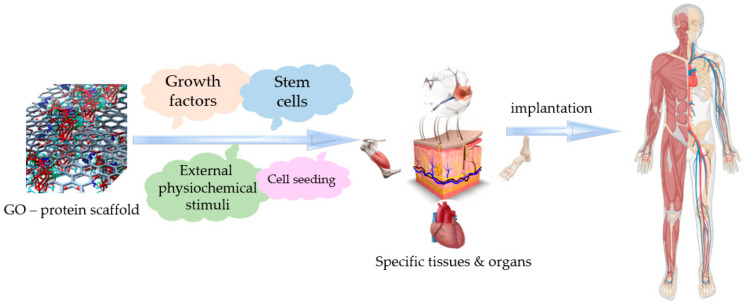
Applications of graphene oxide–protein-based scaffolds in tissue engineering.

The foremost objective of tissue engineering is to design and construct a 3D pattern that mimics the framework and biological functions of the natural extracellular matrix and sustains cell adhesion, growth, and differentiation to manufacture new tissue (Figure 4). There are several parameters that build the path from a theoretical concept to a practical approach when it comes to such biomedical applications, namely, the adsorption affinity of the protein to the carbonaceous matrix and its configuration and biochemical activity in the adsorbed state. The final features of the graphene biocomplex depend on interactions with proteins, which determines the bioactivity and biocompatibility of the nanomaterial.

A more recent approach for the development of advanced nanomaterials with bioactive properties in the field of tissue engineering comprises the seeding of stem cells on suitable scaffolds [148,149,150]. Stem cells represent a special type of cell that can mostly be isolated from the placenta, adipose tissue, bone marrow, and dental pulp and have the ability to grow and differentiate into numerous classes of specialized cell lines. Thus, the seeding of such self-repairing cells on a suitable platform can lead to the formation of new micro-engineered tissue by employing a suitable environment and also by using specific biological signals to help the cells to proliferate and identify the implantation site [151,152,153,154].

Considering this principle, Rezaei et al. [155] fabricated a nanocomposite hydrogel based on collagen and GO functionalized with chitosan as a scaffold for neural stem/precursor cells (NS/PCs). Collagen, by its nature, is a hydrophobic molecule, and thus, numerous polar functional groups introduced by GO significantly improve the water affinity of the hydrogel, which will consequently favor cell adhesion. Using SEM analysis, the authors demonstrated that the nanocomposite hydrogel provided a favorable environment for stem cells to proliferate and grow. In addition, a very interesting outcome was the ability of the cells to migrate along the hydrogel. This behavior represents an important advantage that can be harnessed for the development of bioactive platforms for the treatment of various neurodegenerative disorders.

Following the same principle, Ligorio and collaborators [156] proposed an injectable nanocomposite hydrogel as an innovative platform for the delivery of nucleus pulposus (NP) cells for the repair of intervertebral disc tissue. The self-assembling hydrogel formulation was based on FEFKFEFK peptide and GO. The presence of GO flakes did not affect the β-sheet conformation of the peptides but induced an increase in the shear rate and mechanical properties of the bulk material due to electrostatic interactions. All of these parameters are strongly related and can be altered as a function of the pH and peptide concentration. As previously demonstrated, in this case, GO also improved the viability of NP cells, which showed increased metabolic activity after 7 days of culture. In addition, the similar morphology to the native NP and good injectability of the material make it a suitable candidate as a regenerative therapeutic platform for intervertebral disc repair.

The continuous need for advanced formulations with increased biocompatibility and close resemblance to the natural tissue morphology motivated the use of decellularized extracellular matrix as an active material for the development of tissue engineering scaffolds owing to its protein-based morphology. A recent study [157] proposed the decellularized small intestine submucosa (SISMA) as a biomaterial for the development of a nanocomposite hydrogel. In order to obtain a mechanically stable construct, SISMA was chemically modified with photo-crosslinkable methacryloyl groups. The incorporation of GO into the formulation demonstrated the potential of this biomaterial for tissue engineering applications that require electroconductive properties, such as neuromuscular tissue.

In addition to the aforementioned nanocomposite formulations, GO can be used as a matrix for the differentiation of stem cells [158]. Multilayer GO was chemically modified with four peptide sequences in order to generate a biocompatible substrate for the osteogenic differentiation of mesenchymal stem cells derived from human Wharton’s jelly [159]. Cell viability tests demonstrated that the bioconjugate nanoplatform promoted and enhanced the viability of Wharton’s jelly mesenchymal stem cells and also stimulated osteogenic differentiation.

Due to the limitations imposed by nanocomposite materials chemistry, recently, researchers tried to develop more functional materials in order to improve on the classical formulation and tried to strengthen the bond between the components with respect to graphene oxide and proteins in order to obtain innovative materials as regenerative platforms. Shen et al. [160] applied carbodiimide chemistry in order to functionalize graphene oxide nanosheets with BSA. They obtained a water-soluble bioconjugate and did not alter the biological activity of the protein. Moreover, a recent study developed by Di Santo et al. [161] proposed a complex between GO and human plasma protein (HP) as a strong tool to develop personalized nanoplatforms for in vitro diagnostics.

Graphene oxide has recently stood out as an effective nanomaterial with various applications in tissue engineering and regenerative medicine. Notably, the conjugation of this carbonaceous structure with biomolecules such as proteins or peptides has shown that the resulting complexes have strong potential as support materials for cell-based therapies. However, in addition to the numerous advantages granted by protein–carbonaceous nanocomplexes, there are specific challenges that need to be considered due to the different natures of each component [162,163,164].

When it comes to graphene oxide, physicochemical characteristics such as morphology, shape, dimensions, functional groups present on the basal plane, and the tendency to agglomerate strongly influence the biological activity and the interactions with living cells. Pandit et al. [165] demonstrated that there is a considerable difference between surface-functionalized GO and edge-functionalized GO in terms of protein affinity. It has already been proved that cell viability and proliferation are enhanced by graphene oxide; nevertheless, nanocomposite constructs used as tissue engineering scaffolds need to biodegrade as the new tissue is formed. In terms of biocompatibility, graphene-based materials show dose dependency. After degradation, thin carbon-based nanoflakes with sharp edges can disrupt the integrity of the cellular membrane through a physical mechanism and remain in the organism for long periods of time; thus, low concentrations of GO need to be used [166]. Despite that, Li et al. [167] proved that GO can be partially degraded into small fragments in human blood plasma.

Interactions between GO and proteins develop between the functional groups of both constituents, and thus, in the case of the protein component, several conformational modifications may occur. Hampitak et al. [98] investigated the interactions of BSA with graphene-based structures through quartz crystal microbalance (QCM) techniques and concluded that the hydrophobic character and the functionalization degree of GO strongly influence the orientation, denaturation, and mass absorption of BSA protein.

## 6. Conclusions and Future Considerations

As the need for more biocompatible and mechanically stable scaffolds for regenerative medicine increases, a deeper understanding of the properties, interactions, and synergistic effects of the materials is required. The use of graphene derivatives in the synthesis of 3D scaffolds has had a revolutionary impact in the field of tissue engineering, providing exceptional mechanical properties and organ-biomimicking features. The strong ability of GO/rGO nanostructures to direct stem cell differentiation to specific tissues such as bone, nerve, and cardiac cells is exciting. The presence of GO/rGO nanomaterials in protein scaffolds clearly improves the mechanical properties. Having a large specific surface area and distinctive surface chemistry, GO derivatives engage with proteins and peptides to form strong interconnections. Interfacial interactions can be further adjusted through functionalization by covalent or non-covalent attachment. Furthermore, depending on the tissue to be cultured, the mechanical properties and morphology of the scaffolds can be modulated during the synthesis step. In addition to reinforcing effects, the presence of GO nanostructures creates nanopores, which allow nutrient diffusion and waste discharge during tissue regeneration, replicating in vivo conditions. Additionally, high electrical conductivity is another remarkable property that supports the use of graphene derivatives scaffolds for cardiac and nerve tissue culture. However, protein–GO/rGO interactions need to be evaluated, as they may affect the protein conformation and stability, with a significant influence on the biological activity. Further studies to elucidate these interactions and binding mechanisms will open novel pathways in developing organ-specific scaffolds for tissue engineering.

## Figures and Tables

**Table 1:** Tissue engineering applications of GO-protein/peptide bioconjugates.

Biocomplex	Formulation	Tissue Engineering Application	Conclusions	Ref.
GO/peptide FEFKFEFK (F: phenylalanine; K: lysine; E: glutamic acid)	Hydrogel	Nucleus pulposus (NP) regeneration	GO provides mechanical reinforcement to the hydrogel, facilitates cell adhesion, and can also load and deliver growth factors.	Ligorio et al. [106]
GO/acellular cartilage extracellular matrix	Scaffold	Cartilage tissue regeneration	Composite scaffolds showed increased biocompatibility and reduced inflammatory response after implantation and favored cartilage tissue regeneration.	Gong et al. [107]
GO/gelatin	Aerogel	Skin tissue regeneration/wound healing	The nanocomposite aerogel exhibits hemostatic activity and clogging properties suitable for wound dressing applications.	Borges-Vilches et al. [108]
GO/gelatin	Hydrogel	Tissue adhesive and regeneration	The synthesized formulation showed increased biocompatibility, high mechanical properties, and the ability to promote fibroblast proliferation.	Ryu et al. [109]
GO/poly L-alanine	Thermogel	Adipose tissue engineering	GO–peptide thermogel favored cell differentiation of seeded tonsil-derived mesenchymal stem cells. GO improved cell adhesion and acted as a carrier for growth factors.	Patel et al. [110]
GO/gelatin	Hydrogel	Skeletal muscle regeneration	Nanocomposite hydrogel favored the instinctive myogenic differentiation of C2C12 myoblasts without the aid of external growth factors.	Kang et al. [111]
GO/GelMA/PCL	Nanofibers	Peripheral nerve regeneration	rGO improved the mechanical and electrical properties of the formulation and, at lower concentration of about 0.25–0.5 wt%, enhanced Schwann cell (RSC96) proliferation.	Fang et al. [112]
GO/gelatin/alginate	Nanofibrous scaffolds	Skin tissue engineering	The hybrid electrospun scaffold comprising carboxylated GO exhibited increased biocompatibility and proved to be an appropriate environment for cell adhesion and proliferation.	Ghitman et al. [113]
GO/collagen/PCL/chitosan	Electrospun scaffold	Bone tissue engineering	The concentration of GO within the polymeric scaffold strongly influenced cell adhesion and proliferation, and the nanocomposite with a high ratio of GO showed the most increased osteogenic activity.	Aidun et al. [114]
GO/RGD peptide/PLGA	Nanofibrous mat	Vascular tissue engineering	The 3D structure of the electrospun network was similar to the ECM. The presence of both GO and RGD sequence favored cellular adhesion and proliferation.	Shin et al. [115]

## Data Availability

Data are contained within the article.
